# Investigation of psychological factors related to compassion fatigue, burnout, and compassion satisfaction among nurses

**DOI:** 10.1186/s12912-023-01174-3

**Published:** 2023-01-11

**Authors:** Aslı Yeşil, Şehrinaz Polat

**Affiliations:** 1 Faculty of Humanities and Social Sciences, Department of Psychology, Bursa Technical University, Bursa, Türkiye; 2 Faculty of Nursing, Istanbul University, Istanbul, Türkiye

**Keywords:** Compassion fatigue, Burnout, Compassion satisfaction, Coping strategies, Depression; anxiety

## Abstract

**Background:**

While compassion fatigue is evaluated positively in nurses, compassion fatigue and burnout are undesirable from the viewpoint of professionals, service providers, institutions and ultimately society. It is necessary to identify the factors that lead to undesirable results and to reduce their effects. This study aimed to investigate nurses’ levels of compassion fatigue, compassion satisfaction, burnout, various psychopathological symptom levels, coping skills, and the relationship between them.

**Methods:**

This was a descriptive cross-sectional study. The participants were 356 nurses working in tertiary university hospitals in Istanbul (Türkiye). The Healthcare Professional Information Form, ProQOL-IV, Brief Symptom Inventory, and the Coping Orientations to Problems Experienced scale were used to collect data. Descriptive statistics, correlation analyses, and regression models were used to analyze the data.

**Results:**

According to the findings, low-level burnout, moderate-high compassion satisfaction, and low-moderate compassion fatigue symptoms were detected. Low-level anxiety, depression, somatization, hostility, and negative self-esteem were found. According to the results of regression analysis, mental disengagement and planning coping strategies positively affect the synergy of compassion fatigue (*p* < 0.05). Turning religion and restraint coping have a positive effect on compassion fatigue (*p* < 0.05). While depression has a positive effect on burnout, nurses’ positive reinterpretation and growth strategy is effective in coping with burnout (*p* < 0.05). Positive reinterpretation and growth coping strategies are also effective in increasing job satisfaction (*p* < 0.05).

**Conclusions:**

Nurses showing somatization symptoms are risk factors for compassion fatigue, and nurses showing depression symptoms are risk factors for burnout, so they should be closely monitored and should be given support. Mental disengagement and planning coping strategies can reduce compassion fatigue, and positive reinterpretation and growth methods can reduce burnout and increase compassion satisfaction. It may be useful to provide counseling and training for nurses to use the right coping methods.

## Introduction

Nurses are among the basic service providers of health services. Nurses, due to their duties, deal with the difficult treatment processes of the patients and witness the fears, helplessness, stressful and traumatic experiences of the patients. The nursing profession requires constant communication with people. In this process, the information obtained about the patients’ conditions while performing their care and treatment enables them to provide the best service to their patients [1–10]. Nurses’ long-term therapeutic relationships with patients and patients’ relatives not only cause them to be exposed to more stress but also risk compassion fatigue [3]. In the process of bonding with patients, nurses can perceive positive or negative emotions that eventually lead to compassion satisfaction and compassion fatigue [11]. While an inadequate working environment can be a risk factor for professional compassion fatigue, the satisfaction of being able to serve in an inadequate working environment for another professional may also occur [12].

Compassion fatigue may occur when the nurse cannot control her patients' emotions, is repeatedly exposed to stress and is unable to alleviate the patient's pain. Compassion fatigue decreases in the care capacity. Nurses may experience traumatic stress symptoms or posttraumatic stress disorder due to the intensity of compassion fatigue. Secondary traumatic stress is stress that occurs as a result of wanting to help or helping a person who is suffering or traumatized [1, 3, 5–10, 13].

Although comparison fatigue is similar to burnout, there is a difference between them [6, 7]. Burnout develops slowly and includes progressively worsening. Compasion differs from stress with symptoms such as a gradual increase in workload, lack of success, loss of idealism, and unsupportive work environment. Compasion stress can occur suddenly, and there is a sense of helplessness and confusion [6, 7, 14]. Burnout and compassion stress have negative features related to work. Both secondary trauma and burnout can cause difficulties in the life of the employee [12]. Nurse burnout is an occupational hazard that affects nurses, patients, organizations and society in general [15]. Burnout, patient care and outcomes (quality of care, patient safety, adverse events, patient dissatisfaction, medical errors, infections, pressure sores, patient falls), employee outcomes and job performance (organizational commitment, nurse productivity, turnover, job performance, general health, sickness absence, etc.) [15–18].

Compasion satisfaction is the positive feeling that about the task being done well, feeling of satisfaction from helping another, and the ability to make a positive contribution to the work environment or society [12, 14]. The feeling that the professional has fulfilled the requirements of his/her profession, the feeling of satisfaction experienced, reduces the professional’s secondary traumatic stress [19]. A sense of satisfaction is an important component in the development of compassion fatigue resilience [19]. Compassion satisfaction can be considered a protective factor from occupational psychological risks [20]. There was a negative relationship between burnout and compassion satisfaction [18, 21]. According to Yu and Gui (2022), while compassion fatigue and compassion satisfaction directly affect mental health, burnout directly affects physical health [18]. Compasion satisfaction, which is a sense of satisfaction and achievement, and its negative state, compassion fatigue, are critical to nurses' well-being and therefore affect the quality of patient care [11]. Factors that can affect both compasion satisfaction and compassion fatigue and burnout (such as stress and coping methods) need to be well known.

Task-related stress can also cause health workers to experience various mental problems. Mental distress can affect the symptoms of compassion fatigue and burnout, which are a burden of caregiving. Hegney et al. [22]) found a negative relationship between compassion satisfaction and depression; they found a positive relationship between compassion fatigue and burnout and anxiety and a very weak positive relationship between burnout and depression [22]. In a study conducted with nurses during the COVID pandemic, it was found that job satisfaction affects compassion satisfaction, and mental health problems affect compassion fatigue and burnout [23]. As the symptoms of burnout and compassion fatigue increase, the quality of life associated with health decreases. Additionally, as compassion satisfaction increases, the quality of life related to health also increases [24]. The active coping method (Adaptive) positively affects Compass satisfaction, while passive coping creates a risk factor for compassion and burnout. It has been determined that it contributes positively to the development of empathy, resilience, social support, and compassion satisfaction [2]. It was determined that when newly graduated nurses use active coping methods, compassion satisfaction increases, burnout decreases and burnout and secondary traumatic stress are affected when they use passive coping methods [25]. Nurses who work in intensive care units have reported that methods of coping with stress were associated with burnout [26]. As a result, while self-sense satisfaction in nurses is evaluated positively, self-esteem fatigue and burnout are undesirable for employees, service recipients, the institution and ultimately society. It is important to notice and pay attention to prevent potential negative effects and to plan and implement interventions for the factors that cause them. It is necessary to know the factors that may affect them well and to take steps toward them. While there are many studies examining compassion fatigue, compassion satisfaction, and burnout among nurses in the literature, there has been no study in which the levels of affect of these variables from psychological symptoms and coping skills are evaluated together, as far as we know. In this study, we aimed to investigate the relationship between nurses' levels of various psychopathological symptoms and coping skills, compassion fatigue, compassion satisfaction, and burnout levels.

The research questions are stated below.

- 1. Do psychological symptoms affect Empathy Fatigue, Job Satisfaction and Job Burnout in nurses?
- 2. Do nurses' coping methods affect Empathy Fatigue, Occupational Satisfaction and Occupational Burnout?

## Methods

### Study design

This was a descriptive cross-sectional study. A convenience sample method was used in this research. Before starting the research, we contacted the administrator of the tertiary hospital where the study was conducted, and informed consent was obtained from the participants after informing them about the research. The survey was conducted between October 25, 2019, and December 15, 2019. Online questionnaires were distributed to the nurses via Google Forms. Google Form surveys were sent to the e-mail addresses of all nurses by the nursing services management of the hospital. The Google Forms link was sent twice by the nursing services administration (15 days after the first posting). The participants completed the questionnaire and submitted it via their computers, mobile phones, or touchpads. Before the evaluation, the data obtained were prechecked. The questionnaire could be completed only once by each participant. The questionnaires included researcher information, and invalid questionnaires were excluded from in the study.

### Population and samples

The population in this study was 853 nurses working in tertiary university hospitals in Istanbul (Türkiye). The participants were 356 nurses. The sample size was determined to be 266 nurses with the Known Sample Calculation Formula. Asgari The necessary sample size was found to be 266 when it was calculated according to 5% error and 95% confidence levels. The following formula was used to determine the sample size: (n = Nt^2^pq/d^2^ (N-1) + t^2^pq). It was calculated with 50% incidence (p), which is the maximum value in the sample calculation.

The questionnaire was distributed to a total of 853 nurses within the scope of the study to reach a larger group. This study was conducted with 356 volunteer participants. The response rate was determined to be 41.73%.

## Measures

### Brief Symptom Inventory (BSI)

The Brief Symptom Inventory was used to screen for various psychological symptoms [27]. The scale is a Likert-type self-report scale consisting of 53 items and 5 subscales (0 = None, 4 = Too much). Şahin & Durak (1994) conducted a Turkish validity and reliability study [28]. The internal consistency coefficients obtained from the total score of the scale were found to be 0.96, and the internal consistency coefficients of the subscales were found to vary between 0.65 and 0.86 [28]. In our study, the total Cronbach's alpha value of the scale is 0.97, and the Cronbach's alpha value of the subscales is between and.

### The Coping Orientations to Problems Experienced Scale (COPE)

COPE was used to measure copin skills [29]. The scale, which aims to evaluate the coping attitudes used when faced with difficult or overwhelming events or problems in daily life, is a Likert-type self-report scale consisting of 60 questions and 15 subscales. (1 = I would never do such a thing, 4 = Mostly I do). Ağargün et al. [30] conducted a Turkish validity and reliability study [30]. Each subscale provides information about separate coping skills. The high scores obtained from the subscales of the scale give the possibility to comment on which coping attitude is used more by the individual. The subscales are as follows: 1. Positive reinterpretation and development, 2. The mental disengagement, 3. Focusing on the problem and revealing emotions, 4. Useful social support, 5. Active coping, 6. Denial, 7. Religious coping, 8. Joking, 9. Behavioral disregard, 10. Holding back, 11. Use of emotional social support, 12. Alcohol and substance use, 13. Acceptance, 14. suppressing other occupations, 15. Planning. The internal consistency coefficient of the Turkish version was 0.79, and the correlation validity between the subscales of the scale and the total scores ranged between 0.29 and 0.76 [30]. In our study, Cronbach's alpha value of the scales was determined to be 0.86. The Cronbach's alpha values of the subscales of the scale range from 0.27 to 0.87.

### Professional Quality of Life Scale (ProQOL)

The ProQOL-IV (Stamm [14]) was used to measure compassion fatigue, compassion satisfaction, and burnout [14]. The scale is a 6-point Likert-type scale that was developed to measure the effects of providing care to other people, with a total of 30 items consisting of 10 items each (0 = Never, 5 = Very Often). Yeşil et al. [31] conducted a Turkish validity and reliability study [31]. The Cronbach's alpha value of the compassion satisfaction subscale in the Turkish version of the scale is 0.819, and the Cronbach's alpha value of the burnout subscale is 0.622. Cronbach's alpha value of compassion fatigue subscale is 0.835 [31]. In our study, Cronbach’s alpha values of the subscales were 0.86, 0.57, and 0.81 for compassion satisfaction, burnout, and compassion fatigue, respectively.

### Healthcare professional ınformation form

This form was prepared by the researchers and included questions about age, sex, marital status, work style, and workplace stress factors.

### Ethical considerations

All subjects gave their informed consent for inclusion before they participated in the study. Approval was obtained from the hospital where the study was conducted. The first page of the Google survey used in the study included an "Informed voluntary consent form". Just below the voluntary consent form, there were “I Agree” and “I Do Not Accept” buttons to participate in the study. The participant was able to move on to other questions after clicking “I Agree”. When he clicked “I do not accept”, he could not move on to other questions. It was assumed that the nurses who completed the questionnaire by clicking "I agree" agreed to participate in the study voluntarily. All methods were carried out in accordance with the relevant guidelines and regulations. The study was conducted in accordance with the Declaration of Helsinki. Ethical approval was obtained from the Istanbul University Faculty of Medicine Clinical Research Ethics Council for the research, and then the study was started (Project identification code 2019/252, dated 02/22/2019).

### Data analysis

SPSS version 21 software was used to analyze the survey data. Descriptive (frequency, percentage, minimum–maximum-mean scores, and standard deviation) analyses were used to determine the nurses’ demographic characteristics and scale scores. The normality of data distribution was evaluated using the Kolmogorov‒Smirnov and Shapiro‒Wilk tests, and it was found that a normal distribution was not provided. Accordingly, Spearman’s correlation coefficient and simple linear regression analysis (Newey‒West algorithm for providing assumptions) were used in the analysis of relationships between the variables. Finally, multiple linear regression models were performed to explain the effect of the subscales of the BSI and COPE scale on compassion fatigue, compassion satisfaction, and burnout. The statistical results were considered significant at the level of *p* < 0.05.

## Results

Most of the 356 nurses in this study were women (94%, *n* = 336) and married (61%, *n* = 216), and their average age was 35.86 ± 8.92 (range, 19–64) years. The average working time as a nurse was 14 ± 9.23 (range, 1–44) years. More than half of the nurses (51%, *n* = 181) reported that they choose their profession willingly, and 44% (*n* = 157) worked during the day. Descriptive information of nurses about workplace stress factors is included in Table 1. The mean and minimum and maximum values of the variables of the study are shown in Table 2. The correlation values between the Brief Symptom Inventory, the Evaluation of Coping Attitudes, and the Professional Quality of Life Scale subdimensions are given in Table 2. Multiple linear regression models were tested to explore the predictive power of psychological symptoms on ProQOL (Table 3, Model 1; Table 4, Model 1; Table 5, Model 1). Concerning compassion fatigue, psychological symptom variables (somatization) explained 19% of the variance in compassion fatigue (F [5, 350] = 16.46; *p* < 0.001) (Table 3, Model 1). Concerning burnout, psychological symptom variables (depression) explained 21% of the variance in burnout (F [5, 350] = 19.67; *p* < 0.05) (Table 4, Model 1). It was determined that compassion satisfaction was not affected by psychological symptoms.

**Table 1:** Workplace stress factors

Variables	n	%
Task-induced stress (difficulty of the work, workload, high number of patients)		
Yes	301	84.6
No	55	15.4
Role-related stress (task and role definition ambiguities, conflict, lack of professional knowledge and skills)		
Yes	209	58.7
No	147	41.3
Lack of teamwork, intrateam conflicts, problems in relationships		
Yes	191	53.7
No	165	46.3
Total	356	100
Administrative problems (lack of personnel, materials, and communication difficulties with managers)		
Yes	256	71.9
No	100	28.1
To deal with the problems of patients/families other than their medical problems		
Yes	194	54.5
No	162	45.5
Choosing a profession willingly		
Yes	181	50.8
No	175	49.2
Shift predominantly		
Days	157	44.1
Sometimes day, sometimes night	199	55.9
Total	356	100
	Mean	SD (range)
Age	35.86	8.92 (64–19)
Duration of nursing	14.08	9.23 (44–1)

**Table 2:** Descriptive properties of the scales and correlational relationships between variables

Variables	Cronbach alfa	Mean	SD	1	2	3	4	5	6	7	8	9	10	11	12	13	14	15	16	17	18	19	20	21	22
**ProQual** Compassion satisfaction (1)	0.866	30.72	8.845																						
Burnout (2)	0.811	17.848	5.639	-.234^**^																					
Compassion fatigue (3)	0.572	16.14	8.051	-.592^**^	.598^**^																				
**Brief Symptom Inventory**																									
Anxiety (4)	0.917	6.1	7.631	-.129^*^	.383^**^	.374^**^																			
Depression (5)	0.922	8.97	8.785	-.134^*^	.396^**^	.392^**^	.861^**^																		
Negative self-concept (6)	0.902	6.21	7.347	-.114^*^	.400^**^	.389^**^	.866^**^	.845^**^																	
Somatization (7)	0.902	4.77	5.28	-.169^**^	.408^**^	.384^**^	.722^**^	.738^**^	.721^**^																
Hostility (8)	0.818	5.29	4.561	-.134^*^	.365^**^	.372^**^	.767^**^	.751^**^	.758^**^	.712^**^															
**The Coping Orientations to Problems Experienced scale**																									
Positive reinterpretation and growth (9)	0.658	12.72	2.265	316^**^	-.084	-.267^**^	-.149^**^	-148^**^	-.150^**^	-.189^**^	-.173^**^														
Mental disengagement (10)	0.463	9.28	2.27	.031	.056	.015	.200^**^	.169^**^	.190^**^	.128^*^	.185^**^	.119^*^													
Focusing on and venting emotions (11)	0.552	10.77	2.168	.080	.053	-.001	.149^**^	.150^**^	.129^*^	.063	.120^*^	.352^**^	.221^**^												
Seeking social support for instrumetal reasons (12)	0.693	11.46	2.504	.152^**^	.035	-.045	-.070	-.043	-.055	-.072	-.060	.432^**^	.101	.477^**^											
Active coping (13)	0.581	11.66	2.334	.240^**^	-.127^*^	-.221^**^	-.192^**^	-.163^**^	-.198^**^	-.153^**^	-.164^**^	.521^**^	-.056	.243^**^	.385^**^										
Denial (14)	0.62	6.45	2.328	-.064	.229^**^	.129^*^	.234^**^	.208^**^	.225^**^	.253^**^	.249^**^	-.099	.415^**^	.001	-.098	-.201^**^									
Turning to religion (15)	0.854	11.66	3.193	.113^*^	.082	-.043	.073	.086	.084	-.022	.039	.301^**^	.221^**^	.174^**^	.238^**^	.215^**^	.149^**^								
Joking (16)	0.724	7.3	2.514	.017	.124^*^	.002	.140^**^	.092	.126^*^	.126^*^	.095	.054	.361^**^	.028	.191^**^	.018	.417^**^	.168^**^							
Behavioral desengagement (17)	0.582	6.52	2.244	-.148^**^	.256^**^	.223^**^	.329^**^	.281^**^	.345^**^	.298^**^	.251^**^	-.177^**^	.313^**^	.121^*^	-.085	-.332^**^	.517^**^	.060	.268^**^						
Restraint coping (18)	0.27	8.68	2.043	.095	.205^**^	.038	.214^**^	.160^**^	.158^**^	.158^**^	.133^*^	.206^**^	.215^**^	.227^**^	.181^**^	.129^*^	.248^**^	.204^**^	.217^**^	.294^**^					
Seeking social support for emotional reasons (19)	0.597	10.33	2.508	.178^**^	.001	-.039	-.006	.009	-.006	-.021	.004	.345^**^	.131^*^	.444^**^	.597^**^	.335^**^	-.115^*^	.186^**^	.147^**^	-.033	.274^**^				
Taking drugs (20)	0.708	5.22	1.964	-.111^*^	.210^**^	.137^**^	.216^**^	.196^**^	.252^**^	.284^**^	.209^**^	-.279^**^	.215^**^	-.090	-.154^**^	-.158^**^	.459^**^	-.132^*^	.344^**^	439^**^	.141^**^	-.103			
Acceptance (21)	0.511	9.39	2.23	.111^*^	.148^**^	.034	.163^**^	.123^*^	.144^**^	.117^*^	.127^*^	.274^**^	.288^**^	.257^**^	.292^**^	.176^**^	.176^**^	.216^**^	.237^**^	214^**^	.395^**^	.302^**^	.115^*^		
Suppression of competing activities (22)	0.41	9.92	2.093	.173^**^	.129^*^	.014	.054	.049	.039	.047	-.020	.272^**^	.070	.277^**^	.322^**^	.407^**^	.006	.194^**^	.110^*^	.024	.399^**^	.345^**^	.050	381^**^	
Planning (23)	0.578	12.04	2.372	.221^**^	-.175^**^	-.223^**^	-.179^**^	-.154^**^	-.199^**^	-.206^**^	-.213^**^	.496^**^	-.082	.251^**^	.461^**^	.592^**^	-.343^**^	.175^**^	.035	-.360^**^	.107^*^	.352^**^	-.256^**^	178^**^	330^**^

^*^p < .05

^**^p < .001

**Table 3:** Multiple regression analysis summary for BSI and COPE variables predicting compassion fatigue

**Model 1**					
**Independent variables**	**R Square**		**Adjusted R**^**2**^	**t**	**p**
	**β**	**SE**	**β**		
Constant	12.716	0.615		20.668	< .001^*^
Anxiety	0.053	0.121	0.050	0.439	.661
Depression	0.098	0.089	0.107	1.100	.272
Negative self-concept	0.166	0.123	0.152	1.347	.179
Somatization	0.258	0.126	0.169	2.050	.041^*^
Hostility	-0.007	0.148	-0.004	-0.049	.961
R^2^ = 0.191; (F [5, 350] = 16.46; *p* < .001), Harvey test (p) = .113, LM test (p) = .119 Jarque–Bera (p) = .125					
**Model 2**					
**Independent variables**	**R Square**		**Adjusted R**^**2**^	**t**	**p**
	**β**	**SE**	**β**		
Constant	9.630	3.555		2.709	.007^*^
Positive reinterpretation and growth	-0.181	0.245	-0.051	-0.739	.460
Mental disengagement	-0.443	0.212	-0.125	-2.089	.037^*^
Focusing on and venting emotions	0.173	0.228	0.046	0.757	.450
Seeking social support for instrumental reasons	0.301	0.229	0.094	1.312	.191
Active coping	-0.390	0.246	-0.113	-1.584	.114
Denial	0.215	0.236	0.062	0.909	.364
Turning to religion	0.295	0.138	0.117	2.136	.033^*^
Joking	-0.012	0.201	-0.004	-0.061	.951
Behavioral disengagement	0.160	0.257	0.045	0.622	.535
Restraint coping	0.496	0.245	0.126	2.023	.044^*^
Seeking social support for emotional reasons	-0.086	0.220	-0.027	-0.391	.696
Taking drugs	0.523	0.271	0.127	1.928	.057
Acceptance	0.272	0.213	0.075	1.275	.203
Suppression of competing activities	0.422	0.235	0.110	1.792	.074
Planning	-0.539	0.245	-0.159	-2.199	.029^*^
R^2^ = 0.173; (F [15, 340] = 4.27; *p* < .001), Harvey test (p) = .104, LM test (p) = .116 Jarque–Bera (p) = .223					

**Dependent variable: Compassion fatigue**

^*^p < .05

**Table 4:** Multiple regression analysis summary for BSI and COPE variables predicting burnout

**Model 1**					
**Independent variables**	**R Square**		**Adjusted R**^**2**^	**t**	**p**
	**β**	**SE**	**β**		
Constant	15.050	0.423		35.566	< .001^*^
Anxiety	0.016	0.083	0.022	0.192	.848
Depression	0.118	0.061	0.184	1.928	.043^*^
Negative self-concept	0.086	0.085	0.112	1.016	.310
Somatization	0.132	0.086	0.124	1.530	.127
Hostility	0.089	0.102	0.072	0.879	.380
R^2^ = 0.219; (F [5, 350] = 19.67; *p* < .05), Harvey test (p) = .104, LM test (p) = .112 Jarque–Bera (p) = .149					
**Model 2**					
**Independent variables**	**R Square**		**Adjusted R**^**2**^	**t**	**p**
	**β**	**SE**	**β**		
Constant	22.176	2.539		8.736	< .001^*^
Positive reinterpretation and growth	-0.599	0.175	-0.241	-3.429	.001^*^
Mental disengagement	-0.015	0.152	-0.006	-0.096	.924
Focusing on and venting emotions	0.030	0.163	0.011	0.181	.856
Seeking social support for instrumental reasons	0.198	0.164	0.088	1.209	.228
Active coping	-0.206	0.176	-0.085	-1.172	.242
Denial	-0.025	0.169	-0.010	-.146	.884
Turning to religion	0.043	0.099	0.024	0.433	.665
Joking	-0.198	0.143	-0.088	-1.380	.168
Behavioral disengagement	0.272	0.184	0.108	1.478	.140
Restraint coping	0.040	0.175	0.014	0.226	.821
Seeking social support for emotional reasons	0.014	0.157	0.006	0.089	.929
Taking drugs	0.167	0.194	0.058	0.865	.387
Acceptance	0.166	0.152	0.066	1.090	.276
Suppression of competing activities	0.244	0.168	0.091	1.453	.147
Planning	-0.229	0.175	-0.096	-1.308	.192
R^2^ = 0.140; (F [5, 340] = 3.96; *p* < .001), Harvey test (p) = .097, LM test (p) = .110 Jarque–Bera (p) = .202					

**Dependent variable: Burnout**

^*^p < .05

**Table 5:** Multiple regression analysis summary for BSI and COPE variables predicting compassion satisfaction

**Model 1**								
**Independent variables**	**R Square**			**Adjusted R**^**2**^			**t**	**p**
	**β**		**SE**	**β**				
Constant	32.097		0.735				43.643	< .001^*^
Anxiety	-0.216		0.144	-0.187			-1.502	.134
Depression	0.013		0.107	0.013			0.120	.905
Negative self-concept	0.096		0.148	0.080			0.654	.514
Somatization	-0.214		0.150	-0.128			-1.426	.155
Hostility	0.048		0.177	0.025			0.272	.786
R^2^ = 0.062; F [5, 350] = 3.29; *p* < .01, Harvey test (p) = .002, LM test (p) = .034 Jarque–Bera (p) < .001								
**Model 2**								
**Independent variables**		**R Square**			**Adjusted R**^**2**^	**t**		**p**
		**β**	**SE**		**β**			
Constant		15.049	4.021			3.743		< .001^*^
Positive reinterpretation and growth		0.903	0.277		0.231	3.261		.001^*^
Mental disengagement		0.064	0.240		0.016	0.265		.791
Focusing on and venting emotions		-0.186	0.258		-0.046	-0.722		.471
Seeking social support for instrumental reasons		-0.092	0.259		-0.026	-0.355		.723
Active coping		0.072	0.279		0.019	0.259		.796
Denial		0.100	0.267		0.026	0.374		.709
Turning to religion		0.068	0.156		0.025	0.435		.664
Joking		-0.053	0.227		-0.015	-0.233		.816
Behavioral disengagement		-0.324	0.291		-0.082	-1.115		.266
Restraint coping		0.024	0.277		0.006	0.087		.931
Seeking social support for emotional reasons		0.263	0.248		0.074	1.057		.291
Taking drugs		-0.203	0.307		-0.045	-0.662		.509
Acceptance		0.109	0.241		0.027	0.450		.653
Suppression of competing activities		0.266	0.266		0.063	0.998		.319
Planning		0.113	0.277		0.030	0.409		.683
R^2^ = 0.123; (F [5, 340] = 3.18; *p* < .001),Harvey test (p) = .083, LM test (p) = .105 Jarque–Bera (p) = .118								

**Dependent variable: Compasion satisfaction**

^*^p < .05

Multiple linear regression models were tested to explore the predictive power of coping skills on compassion fatigue, burnout, and compassion satisfaction (Table 3, Model 2; Table 4, Model 2; Table 5, Model 2). Concerning compassion fatigue, coping skills variables (mental disengagement, turning to religion, restraint coping, and planning) explained 17% of the variance in compassion fatigue (F [15, 340] = 4.27; *p* < 0.001) (Table 3, Model 2). Concerning burnout, coping skills variables (positive reinterpretation and growth) explained 14% of the variance of burnout (F [5, 340] = 3.96; *p* < 0.001) (Table 4, Model 2). Concerning compassion satisfaction, coping skills variables (positive reinterpretation and growth) explained 12% of the variance of compassion satisfaction (F [5, 340] = 3.18; *p* < 0.001) (Table 5, Model 2).

## Discussion

In this study, the ProQOL (compassion fatigue, compassion satisfaction, and burnout) levels, psychological symptoms, and coping skills of nurses working at a tertiary university hospital were examined. In addition, psychological symptoms and coping skills that affect ProQOL were identified. It was determined that the nurses had low-level burnout, moderate-high compassion satisfaction, and low-moderate compassion fatigue symptoms. This result was also similar to previous studies [20, 21, 23, 32, 33]. Low-level anxiety, depression, somatization, hostility, and negative self-concept were detected.

Very few studies have examined the ProQOL levels and psychological symptoms of nurses in the literature. Hegney et al. [22] found that the anxiety and depression levels of nurses were within the normal range [22]. Zhan et al. [23] also found that 85.60% of participants were healthy [23]. In another study conducted with emergency department nurses, 53.46% of the nurses did not have a depressive tendency in the evaluation of depressive or nondepressive tendencies [34]. In another study conducted with nurses working in a tertiary hospital, low levels of depression and anxiety symptoms were found [35]. Individuals use both emotion-focused and problem-focused coping styles in stressful situations [29, 36]. In this research, it was determined that nurses used problem-focused strategies more intensively than emotion-focused strategies. Al Barmawi et al. [37] found that nurses used seeking social support first, followed by problem-solving and avoidance strategies [37].

Nurses use positive coping styles more than negative coping styles [38]. In another study conducted on nurses’ coping styles and work stress, 65.07% of nurses used adaptive coping skills [39]. The findings obtained from our study could not be compared with previous data because the mean values of coping skills and ProQOL were not included in previous studies examining coping skills and ProQoL [2, 25, 26, 40]. Additionally, the differences in the measurement tools used in the studies caused difficulties in comparing the findings. In our research, it was found that nurses used active coping, planning, and seeking social support for instrumental reasons, which were used at moderate-to-high levels, and used restraint coping and suppression of competing activities at a moderate level. Positive reinterpretation and growth and turning to religion were used at a medium–high level, emotional social support and acceptance skills were used at a moderate level, and denial was used at a low level. Additionally, it was found that focusing on and venting emotions and mental disengagement were used moderately, and behavioral disengagement, taking drugs, and joking were used at low levels.

Individuals use both emotion-focused and problem-focused coping styles in stressful situations [29, 36]. In problem-focused coping, people try to change the source of stress. In emotion-focused coping, when the source of stress cannot be changed, the emotional state causing stress is tried to be reduced, that is, to regulate the emotion [29]. In our study, it can be said that nurses first tried to cope with stressful situations using problem-focused skills and then used emotion-focused coping strategies.

ProQoL is the kind of positive (compassion satisfaction) and negative (compassion fatigue and burnout) feeling that professionals feel as a result of having done their job well [12]. In our study, a positive correlation was found between compassion fatigue and burnout [4, 18, 20, 21, 41–44], and a negative correlation was found between compassion fatigue and compassion satisfaction [20, 41, 42], similar to other studies in the literature. In addition, it was found that there was a negative correlation between compassion satisfaction and burnout [18, 20–22, 41–45].

The concept of compassion fatigue includes burnout and secondary trauma. Both affect mental health [18, 23, 24] and physical health [18]. Previous studies have found a positive correlation between anxiety and depression and a positive correlation between burnout and compassion fatigue [22, 46]. A negative correlation was found between burnout and job satisfaction [47, 48]. In addition, a positive correlation was determined between compassion satisfaction and mental health [18], a negative correlation between compassion satisfaction and compassion fatigue [18], and a negative correlation between anxiety and depression [46]. In our study, it was determined that somatization affected compassion fatigue and that depression affected burnout. Ruiz-Fernandez et al. [24] found that general health symptoms and body pain were related to compassion fatigue and burnout [24].

Coping is a cognitive and behavioral effort used to manage internal and external demands. Intervention to the source of stress is defined as problem-focused coping, and the regulation of emotions caused by a stressful situation is referred to as emotion-focused coping [36]. In our study, it was determined that the use of mental disengagement and planning coping skills were effective in reducing the symptoms of compassion fatigue. The mental disengagement involves several activities [29]. It can be functional because it keeps the person away from stress as soon as it is used. Knowing that these strategies will be used in difficult situations may reduce compassion fatigue. Planning involves finding the best method of coping with a stressor. Planning is a method of problem-focused coping; at the same time, an active problem-solving outline is created. This includes thinking about what to do in situations where stress increases [29]. In previous studies, it was determined that active coping reduced compassion fatigue [2, 25]. Preplanned methods that are ready in the face of emergencies may enable nurses to minimize errors that may arise from themselves and therefore reduce the symptoms of compassion fatigue.

In our study, turning to religion and restraint coping affected the increase in compassion fatigue symptoms. Many people use religion as a source of emotional support under stress and as a tool for positive reinterpretation and growth [29]. Thus, turning to religion can serve as active coping because it involves intrinsically positive reinterpretation [29]. Nurses, who witness and intervene in difficult processes of patients during health care practices, may not be able to positively reinterpret internally for many very difficult situations. It may cause nurses to experience a feeling of being stuck in their internal processes, thereby increasing the symptoms of compassion fatigue. Restraint coping is an active coping method for coping with stress. However, keeping a distance from stress and not acting, not trying, is also a passive way of coping [29]. Standing back and doing nothing may cause feelings of helplessness. According to Varadorojan & Rani (2021), there is a positive correlation between distancing and compassion fatigue [26]. Positive reinterpretation and growth, which is an emotion-focused coping method, aims to cope by trying to manage the emotion affected by the stressor instead of dealing with the stressor [29]. In our study, positive reinterpretation and growth coping approaches were effective in reducing burnout while increasing compassion satisfaction. Positive internal reinterpretation is also an active internal coping process [29]. Positive reinterpretation may reduce burnout, and the feeling of overcoming challenges at work may also increase job satisfaction. Varadorojan & Rani (2021) determined that there was a positive correlation between compassion satisfaction and positive reinterpretation and a negative correlation between burnout and positive reinterpretation [26].

## Limitations

Cross-sectional studies do not allow us to interpret the causal relationships. It was assumed that the people participating in the survey reflected their true feelings when responding to the questions on the scale. It was assumed that the participants responded willingly to the questionnaire and answered the questionnaire correctly and completely. It was assumed that the participants understood the true meaning of the survey questions. Another limitation was that the study was limited to Istanbul Province and a university hospital. The fact that the study was conducted using the convenience sampling method can be counted among the limitations of this study.

## Conclusion

This research provides information about the relationships between nurses' depression, anxiety, somatization, negative self and hostility levels and symptoms of compassion fatigue, burnout and compassion satisfaction. It also provides information about the coping strategies that nurses use for compassion fatigue, burnout and compassion satisfaction symptoms. Nurses showing somatization symptoms are risk factors for empathy fatigue, and nurses showing depression symptoms are risk factors for burnout, so they should be closely monitored, and necessary support should be given. Considering that mental disengagement and planning can reduce empathy fatigue and that positive reinterpretation and development methods can reduce burnout and increase professional satisfaction, it may be beneficial to provide counseling and training for nurses to use correct coping methods. These findings can be used to increase the general well-being of nurses and to develop psychoeducational studies to increase their compassion satisfaction. Increasing the compassion satisfaction of nurses will contribute to the improvement of service quality and job satisfaction and reduce health mistakes.

## Data Availability

The datasets used and/or analyzed during the current study are available from the corresponding author on reasonable request.
